# In vitro and in vivo comparison of MRI chemical exchange saturation transfer (CEST) properties between native glucose and 3‐O‐Methyl‐D‐glucose in a murine tumor model

**DOI:** 10.1002/nbm.4602

**Published:** 2021-08-22

**Authors:** Annasofia Anemone, Martina Capozza, Francesca Arena, Sara Zullino, Paola Bardini, Enzo Terreno, Dario Livio Longo, Silvio Aime

**Affiliations:** ^1^ Molecular Imaging Center, Department of Molecular Biotechnology and Health Sciences University of Torino Torino Italy; ^2^ Center for Preclinical Imaging, Department of Molecular Biotechnology and Health Sciences University of Torino Torino Italy; ^3^ Institute of Biostructures and Bioimaging (IBB) Italian National Research Council (CNR) Torino Italy

**Keywords:** 3‐O‐Methyl‐D‐glucose, CEST, D‐Glucose, glucoCEST, MRI, tumor

## Abstract

D‐Glucose and 3‐O‐Methyl‐D‐glucose (3OMG) have been shown to provide contrast in magnetic resonance imaging‐chemical exchange saturation transfer (MRI‐CEST) images. However, a systematic comparison between these two molecules has yet to be performed. The current study deals with the assessment of the effect of pH, saturation power level (B_1_) and magnetic field strength (B_0_) on the MRI‐CEST contrast with the aim of comparing the in vivo CEST contrast detectability of these two agents in the glucoCEST procedure. Phosphate‐buffered solutions of D‐Glucose or 3OMG (20 mM) were prepared at different pH values and Z‐spectra were acquired at several B_1_ levels at 37°C. In vivo glucoCEST images were obtained at 3 and 7 T over a period of 30 min after injection of D‐Glucose or 3OMG (at doses of 1.5 or 3 g/kg) in a murine melanoma tumor model (n = 3–5 mice for each molecule, dose and B_0_ field). A markedly different pH dependence of CEST response was observed in vitro for D‐Glucose and 3OMG. The glucoCEST contrast enhancement in the tumor region following intravenous administration (at the 3 g/kg dose) was comparable for both molecules: 1%–2% at 3 T and 2%–3% at 7 T. The percentage change in saturation transfer that resulted was almost constant for 3OMG over the 30‐min period, whereas a significant increase was detected for D‐Glucose. Our results show similar CEST contrast efficiency but different temporal kinetics for the metabolizable and the nonmetabolizable glucose derivatives in a tumor murine model when administered at the same doses.

## Graphical abstract

This study investigated the CEST properties of D‐Glucose and 3‐O‐Methyl‐D‐glucose (30MG) in vitro and after intravenous injection in a murine melanoma model. A marked and opposite pH dependence of the CEST contrast was observed in vitro for D‐Glucose and 3OMG, whereas both molecules showed a similar CEST enhancement following the in vivo administration at 7 T, but a reduced detectability at 3 T.

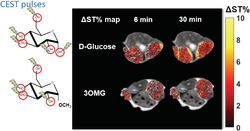

**Abbreviations used**
^[18F]^‐FDG: 2‐Deoxy‐2‐[18F]‐Fluoroglucose
2DG: 2‐Deoxy‐D‐glucose
2OMG: 2‐O‐Methyl‐D‐glucose
3OMG: 3‐O‐Methyl‐D‐glucose
6DG: 6‐Deoxy‐D‐glucose
B0: magnetic field strength
B1: saturation power level
CESL: chemical exchange‐sensitive spin‐lock
CEST: chemical exchange saturation transfer
CT: computed tomography
DGE‐MRI: dynamic glucose enhanced MRI
GlcN: glucosamine
GlcNAc: N‐Acetyl‐Glucosamine
glucoCEST: D‐Glucose chemical exchange saturation transfer
GLUT‐1 and 3: glucose transporters 1 and 3
MRI: magnetic resonance imaging
PBS: phosphate‐buffered saline
PET: positron emission tomography
ROI: region of interest
ST: saturation transfer

## 1 INTRODUCTION

In vivo imaging techniques are currently used to detect tumors and to monitor the response to therapy. Magnetic resonance imaging (MRI) often makes use of contrast agents to augment physiological information to the anatomical resolution of its images.^1^ Nowadays, much attention is devoted to the characterization of tumor metabolism, as it is recognized that knowledge of the metabolic state of cancer cells provides crucial information in the diagnostic assessment of the disease. Glucose, being the primary source of energy, is of course under intense scrutiny as a metabolic tracer.^2^ Positron emission tomography (PET) exploits the increased glucose uptake from tumor cells to report the accumulation of phosphorylated 2‐Deoxy‐2‐[^18^F]‐fluoroglucose ([^18^F]‐FDG), a radioactive glucose analog, and to extract precious information on the ongoing metabolism of the cancer cells.^3^ Indeed, this method is used daily in clinical settings to image primary and metastatic tumors. Major issues that hamper PET modality are associated with the use of radioactive compounds and with the radiation doses that patients receive when PET is carried out in conjunction with computed tomography (CT) to provide the required anatomical resolution. These issues make its application not suitable for all patients (e.g. pregnant women and pediatric application are commonly excluded from PET studies) and quite expensive.^4^

Over the last decade, MRI‐chemical exchange saturation transfer (MRI‐CEST) methods have attracted interest as noninvasive alternatives for studying tumor metabolism and its microenvironment^5^, ^6^, ^7^; and CEST endogenous contrast has been widely explored in preclinical and clinical studies.^8^ Magnetization transfer between exchangeable protons from amine,^9^, ^10^ amide^11^, ^12^ or hydroxyl groups^13^, ^14^ resonating at 1–3 ppm downfield from the water resonance can be exploited for CEST applications. Much attention has been devoted to the use of glucose as a MRI‐CEST agent as it would yield a significant cost reduction and an increase in safety and accessibility in comparison with PET, yet preserving and potentially improving its specificity for tumor characterization and evaluation of response to therapy.

Exploiting OH exchangeable protons as the source of the MRI‐CEST effect, natural glucose proved its usefulness in cancer detection. A first demonstration was provided when D‐Glucose chemical exchange saturation transfer (glucoCEST) showed enhanced contrast in two human breast cancer cell lines orthotopically implanted in mice, in agreement with FDG‐PET enhancement.^15^ Later on, comparing the glucoCEST signal with [^18^F]‐FDG autoradiography in a human colorectal tumor mouse xenograft model, provided further support of the view that glucoCEST is specific and provides as sensitive a measure as FDG uptake.^16^ Moreover, dynamic glucose enhanced MRI was also used to study malignant brain tumor and blood–barrier breakdown at a clinical level.^17^, ^18^ It was also shown that the sensitivity of mapping the glucose concentration in the brain could be increased by assessing the OH proton exchange by means of spin‐lock MRI.^19^, ^20^ Recently, in a detailed study of the exchange rate constant of glucose hydroxyl groups, optimal parameters for in vivo glucoCEST/chemical exchange‐sensitive spin‐lock (CESL) detection at clinical and ultrahigh field strengths were reported.^21^, ^22^, ^23^

However, compared with [^18^F]‐FDG, glucose is rapidly metabolized through glycolysis, thus causing uncertainty about its concentration in tumor cells, with a consequent decrease of the CEST signal. For this reason, glucose analogs that are not metabolized upon their uptake into tumor cells are being considered as possible alternatives for MRI‐CEST procedures. For instance, analogs such as 2‐deoxy‐D‐glucose (2DG), dextran, sucralose, sucrose, glucosamine (GlcN) and N‐acetyl‐glucosamine (GlcNAc), which are phosphorylated as glucose,^24^, ^25^, ^26^, ^27^, ^28^ and analogs that do not undergo phosphorylation (e.g. 2‐O‐Methyl‐D‐glucose [2OMG], 3‐O‐Methyl‐D‐glucose [3OMG] and 6‐deoxy‐D‐glucose [6DG]), have been studied intensely.^29^, ^30^, ^31^ Among the first group, GlcN is considered noteworthy for its excellent safety profile^28^ and for the presence in its structure of the amino peak that yields a CEST signal that is more shifted than the hydroxyl ones from water, thus making the CEST response more efficient, in particular at clinical fields. In the second group, 3OMG appears an interesting alternative as it enters the cells through the glucose transporters (GLUT‐1 and 3). 3OMG displayed higher and longer lasting CEST signal compared with D‐Glucose for the same type of murine tumor,^32^ but detailed studies of its toxicology are still lacking, even though it did not induce any physiological or behavioral changes for various dosages in mice and rats.^33^

Although successful proofs of concept have been provided and clinical investigations have been reported for D‐Glucose,^20^, ^34^, ^35^ to date no comprehensive studies investigating D‐Glucose and 3OMG on the same tumor model and under the same experimental conditions are available. The aim of the current study is to systematically evaluate the effects of pH, saturation power level (B_1_) and magnetic field strength (B_0_) on the generation of CEST contrast of D‐Glucose and 3OMG for a proper comparison between the two molecules. Taking into account that the two molecules are characterized by dissimilar metabolic fates, we hypothesized that a different CEST contrast could be observed under the same experimental conditions.

Moreover, we assessed their in vivo capability to provide contrast when administered to a murine melanoma model upon an intravenous (i.v.) injection at two different doses (1.5 and 3 g/kg) and at two field strengths (3 and 7 T).

## 2 METHODS

### 2.1 Chemicals

D‐Glucose and 3OMG powder for in vitro studies were obtained from Sigma‐Aldrich (Milan, Italy). Solutions of D‐Glucose and 3OMG for in vitro studies were prepared in 10 mM phosphate‐buffered saline (PBS 1X). Glucose or 3OMG injectable solution for in vivo studies was prepared by dissolving the powder in saline solution to obtain a 3 M solution at neutral pH (7.4). The solution was then filtered with 200‐nm membrane filters to preserve the suspensions from bacterial contamination. 3OMG powder for in vivo studies was kindly provided by Almac (UK).

### 2.2 Phantom preparation

Phantoms containing different vials of 10 mM PBS 1X of D‐Glucose and 3OMG were prepared starting from a 20 mM solution. Each solution was then titrated to reach the intended pH values of 7.4, 7.0, 6.8, 6.6, 6.4, 6.2 and 6.0.

### 2.3 Tumor animal model

#### 2.3.1 Cell culture

B16‐F10 (mouse melanoma cells) were obtained from American Type Culture Collection. B16‐F10 cells were cultured in EMEM supplemented with 10% FBS, 100 μg/mL penicillin and 100 μg/mL streptomycin. The cells were grown at 37°C in a humidified atmosphere containing 5% CO_2_. At confluence, cells were detached by adding 1 mL of Trypsin–EDTA solution (0.25%_w/v_ Trypsin, 0.53 mM EDTA). EMEM, FBS and Trypsin were purchased from Lonza (Verviers, Belgium). The penicillin–streptomycin mixture was purchased from Sigma Chemical Co. (St. Louis, MO, USA).

#### 2.3.2 Subcutaneous implantation of tumor cells in mice

Eight‐week‐old male C57BL/6 mice (Charles River Laboratories, Calco, Italy) were inoculated with 5.0 x 10^5^ B16‐F10 melanoma cells in both flanks 10 days before imaging. Mice were maintained under specific pathogen‐free conditions in the animal facility of the Centre for Preclinical Imaging, University of Turin, and treated in accordance with the University Ethical Committee and European guidelines under directive 2010/63.

B16‐F10 tumor‐bearing mice were randomly divided into two cohorts (one for each molecule of D‐Glucose and 3OMG) of four groups (each containing 3–5 mice) to investigate the elicited CEST contrast after i.v. injection of D‐Glucose or 3OMG at the two doses of 1.5 and 3 g/kg and at the two main magnetic fields of 3 and 7 T.

Before imaging, mice were anesthetized with isoflurane and placed on the MRI bed and monitored through an air pillow located below the animal (SA Instruments, Stony Brook, NY, USA). The tail vein was cannulated with a catheter with a 27‐gauge needle.

### 2.4 MRI‐CEST protocol and analysis

In vitro Z‐spectra were acquired on a 3‐T Bruker BioSpec scanner equipped with a 1H quadrature coil and on a 7‐T Bruker Avance 300 scanner equipped with a micro 2.5 imaging probe. The experiments were carried out at 37°C by irradiating the sample with a single continuous wave presaturation block pulse (1.0, 2.0 and 3.0 μT) applied for 5 s. The saturation frequency offset was varied from 10 to −10 ppm with a frequency resolution of 0.1 ppm. MR images were acquired using a spin‐echo RARE sequence (TR/TE/NEX/rare factor: 10.0 s/5.4 ms/2/64) with centric encoding, field of view = 3 x 3 cm, slice thickness = 2 mm and matrix = 64 x 64.

In vivo studies were carried out on a 7‐T Bruker Pharmascan scanner (Bruker Biospin, Ettlingen, Germany) equipped with a 30‐mm 1H quadrature coil and on a 3‐T Bruker BioSpec scanner equipped with a 30‐mm 1H quadrature coil. After the scout image, T_2w_ anatomical images were acquired with a RARE sequence and the same geometry was used for the following CEST experiments. The glucoCEST (before and after injection of D‐Glucose or 3OMG) images were obtained by irradiating the animal with a single continuous wave presaturation block pulse of 2 μT applied for 5 s. The CEST acquisition protocol was kept constant across B0 fields and molecules, with Z‐spectra sampled with 61 frequency offsets over a range of ±10 ppm and with a step size of 0.2 ppm over a range of ±6 ppm. CEST images were recorded with a single‐shot Fast Spin Echo sequence with centric encoding (TR 6.0 s, TE 4.7 ms, Rare Factor 64, field of view = 3 cm x 3 cm, slice thickness = 2 mm, matrix = 64 x 64). CEST images were acquired before and every 6 min following the injection up to 32 min.

In‐house MATLAB scripts (MathWorks, Natick, MA, USA) were used to process all the CEST images. Firstly, anatomical and Z‐spectrum images were segmented by using an intensity‐threshold filter; secondly, Z‐spectra were interpolated on a voxel‐by‐voxel basis by smoothing splines^36^ to identify the correct position of the bulk water and remove artifacts arising from B_0_ inhomogeneity. Then the interpolated Z‐spectrum was shifted to make the bulk water resonance match the zero frequency and corrected intravoxel saturation transfer (ST) effects were calculated with asymmetry analysis.^37^ To remove CEST effects arising from noisy data, a second filter was applied by calculating the coefficient of determination (R^2^) for the interpolating curve, and by taking into consideration the signal‐to‐noise ratio of single voxels (noisy Z‐spectra present low R^2^ values); in the ST% calculation, only voxels with high R^2^ (>0.97) were considered. The ST effect for glucose or 3OMG was estimated at 1.2 ppm (for in vitro glucose data only, the contrast was calculated at 0.8 ppm) from the expression:

$$\mathrm{ST}=\left(S\left(-1.2\;\mathrm{ppm}\right)-S\left(1.2\;\mathrm{ppm}\right)\right)/S_{0},$$

where S_0_ is the signal intensity at −10 ppm and S(±1.2 ppm) is the signal intensity at ±1.2 ppm.

Results are reported as:

$$\mathrm{\Delta ST}\%=\left[\left(\mathrm{ST}\;\text{post injection}–\mathrm{ST}\;\text{preinjection}\right)\;x\;100\right].$$

The fraction of enhanced pixel reports on the percentage of pixels showing a ΔST% greater than zero in the manually defined tumor region of interest (ROI).

### 2.5 Statistical analysis

GraphPad Prism 7 software (GraphPad Inc., San Diego, CA, USA) was used for statistical analysis. Data are presented as mean ± SD unless otherwise stated. One‐way ANOVA analysis and Dunnet's multiple comparison test were used to test for statistically significant differences between the ΔST measurements over time. For all tests, *p* less than 0.05 was considered statistically significant.

## 3 RESULTS

### 3.1 In vitro MRI‐CEST characterization

**FIGURE 1:**
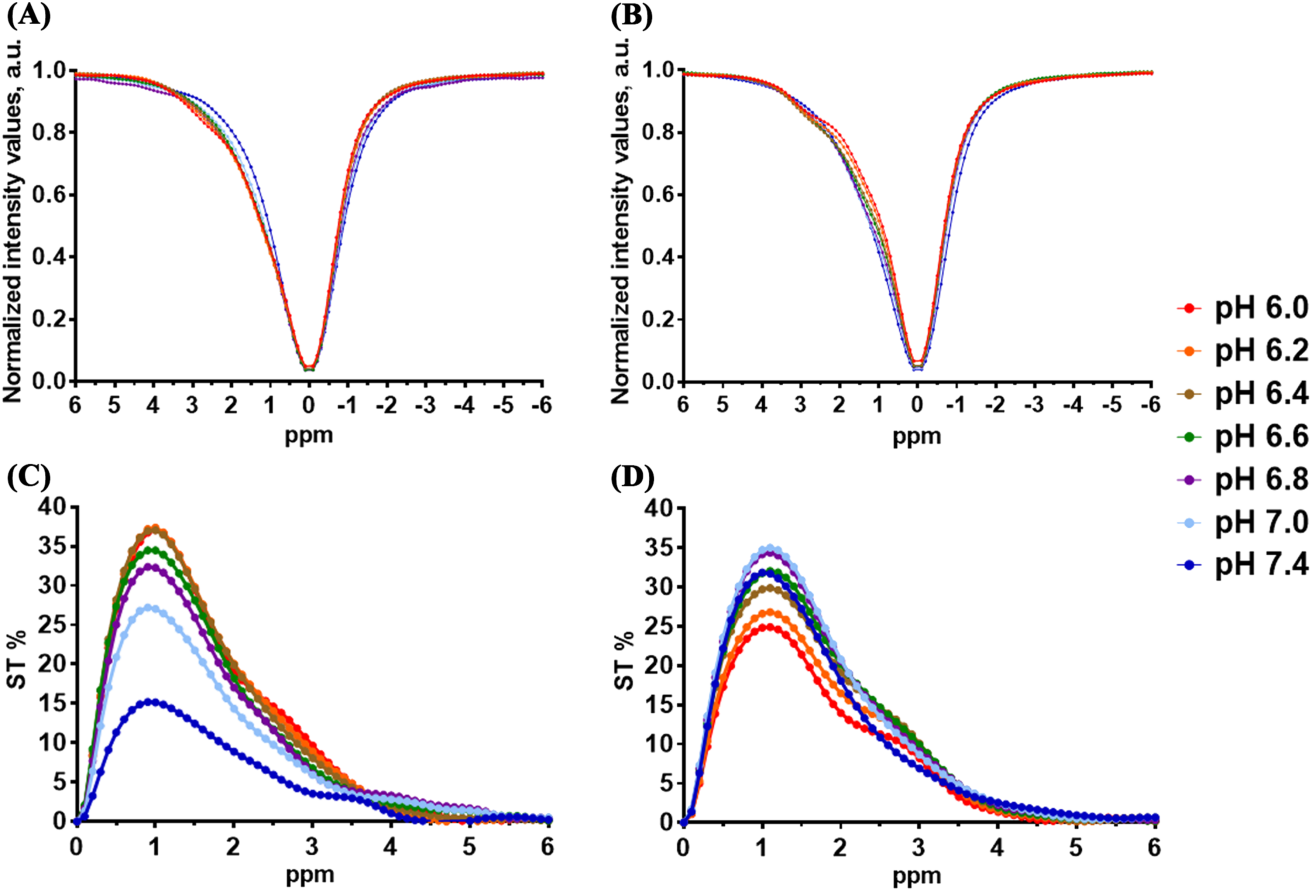
D‐Glucose displayed the higher chemical exchange saturation transfer (CEST) effect at acidic pH values whereas 3‐O‐Methyl‐D‐glucose (3OMG) showed higher CEST contrast at neutral pH and at 7 T. Z‐spectra and percentage change in saturation transfer (ST%) effect plot of 20 mM (A and C) D‐Glucose (calculated at 0.8 ppm) and of (B and D) 3OMG (calculated at 1.2 ppm) solutions containing 10 mM of phosphate buffer as a function of pH values acquired at 37°C with a 7 T scanner (B_1_ = 3.0 μT)

**FIGURE 2:**
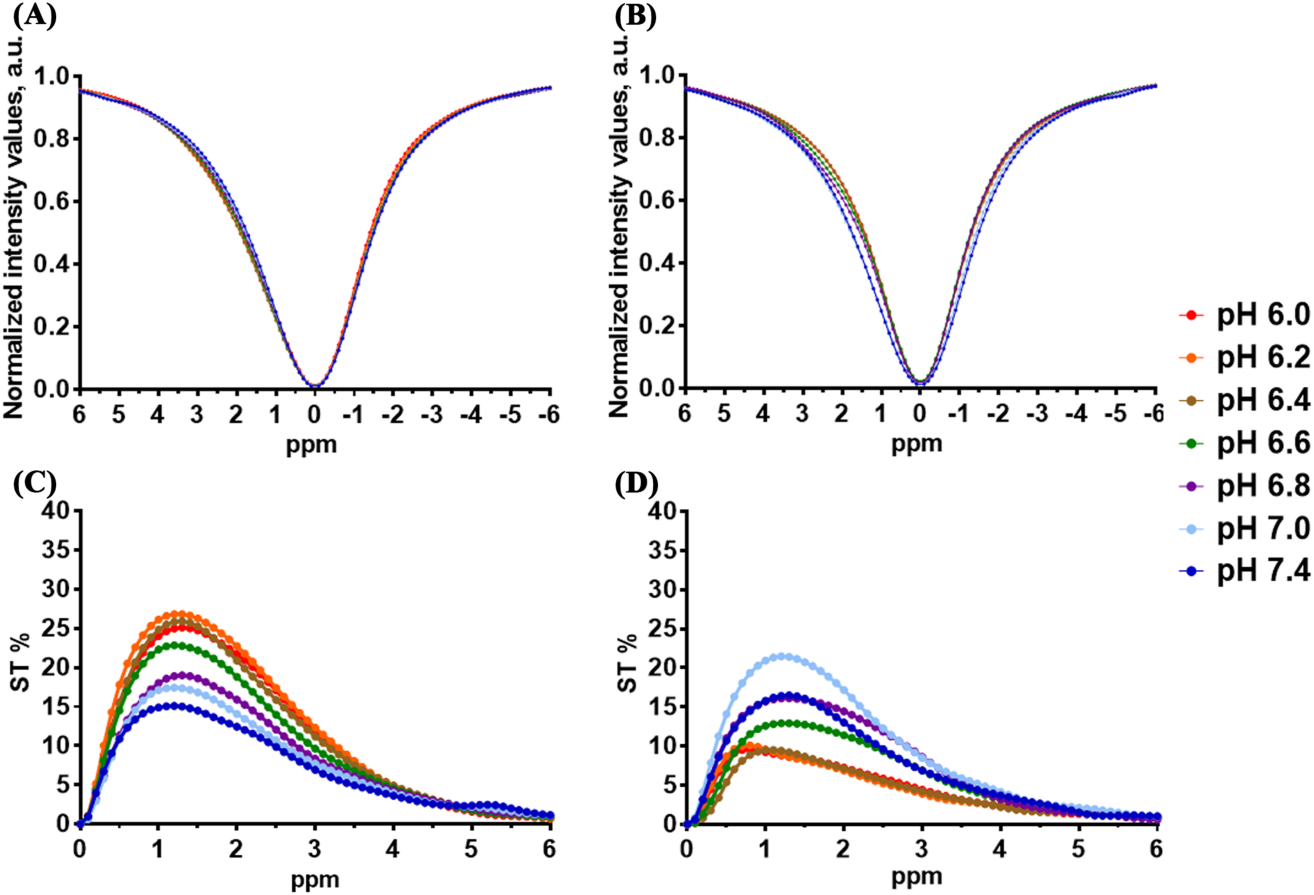
At 3 T, lower chemical exchange saturation transfer (CEST) effects were detected for both D‐Glucose and 3‐O‐Methyl‐D‐glucose (3OMG) with a different pH trend. Z‐spectra and percentage change in saturation transfer (ST%) effect plot of 20 mM (A and C) D‐Glucose (calculated at 0.8 ppm) and of (B and D) 3OMG (calculated at 1.2 ppm) solutions containing 10 mM of phosphate buffer as a function of pH values acquired at 37°C with a 3 T scanner (B_1_ = 3.0 μT)

To assess the magnetic field and pH‐dependent properties of D‐Glucose and 3OMG, phantoms containing the solutions at different pH values were investigated. Z‐spectra were acquired on two MRI scanners, namely, on a high field 7‐T scanner and on a preclinical scanner working at a clinical field of 3 T. The chemical shift (from water resonance) of 0.8 ppm for D‐Glucose and of 1.2 ppm for 3OMG were chosen as they correspond to the highest CEST signals. The ST effect at 7 T measured at 37°C for 20 mM D‐Glucose solution at different pH values using 3.0 μT of saturation pulse is shown in Figure 1. The CEST effects calculated from the asymmetry analysis in the B_0_‐corrected Z‐spectra generated by D‐Glucose reached 36% between pH values of 6.4 and 6.0 (Figure 1A,C). The CEST effect appears markedly pH‐dependent, as at neutral pH a net decrease in the ST effect is clearly observed. Figure 1B shows the B_0_‐corrected Z‐spectra generated by solutions containing 20 mM of 3OMG obtained by using the same acquisition parameters reported above for D‐Glucose‐containing solutions. Compared with D‐Glucose, the 3OMG signal reached a higher CEST effect at neutral pH (around 35% between pH 7.0 and 6.8) and decreased at acidic pH values (25% of the ST effect at pH 6.0; Figure 1D). At 3 T, broader Z‐spectra and lower contrasts were detected (Figure 2A,B); the highest ST effect for D‐Glucose (close to 25%) was observed between pH 6.0 and 6.4 (Figure 2C), as detected in the high field experiment. 3OMG, under the same acquisition conditions, showed lower ST effects (close to 10%) compared with D‐Glucose between pH 6.0 and 6.4 (Figure 2D), whereas the highest effect (20%) was detected at neutral pH (7.0).

**FIGURE 3:**
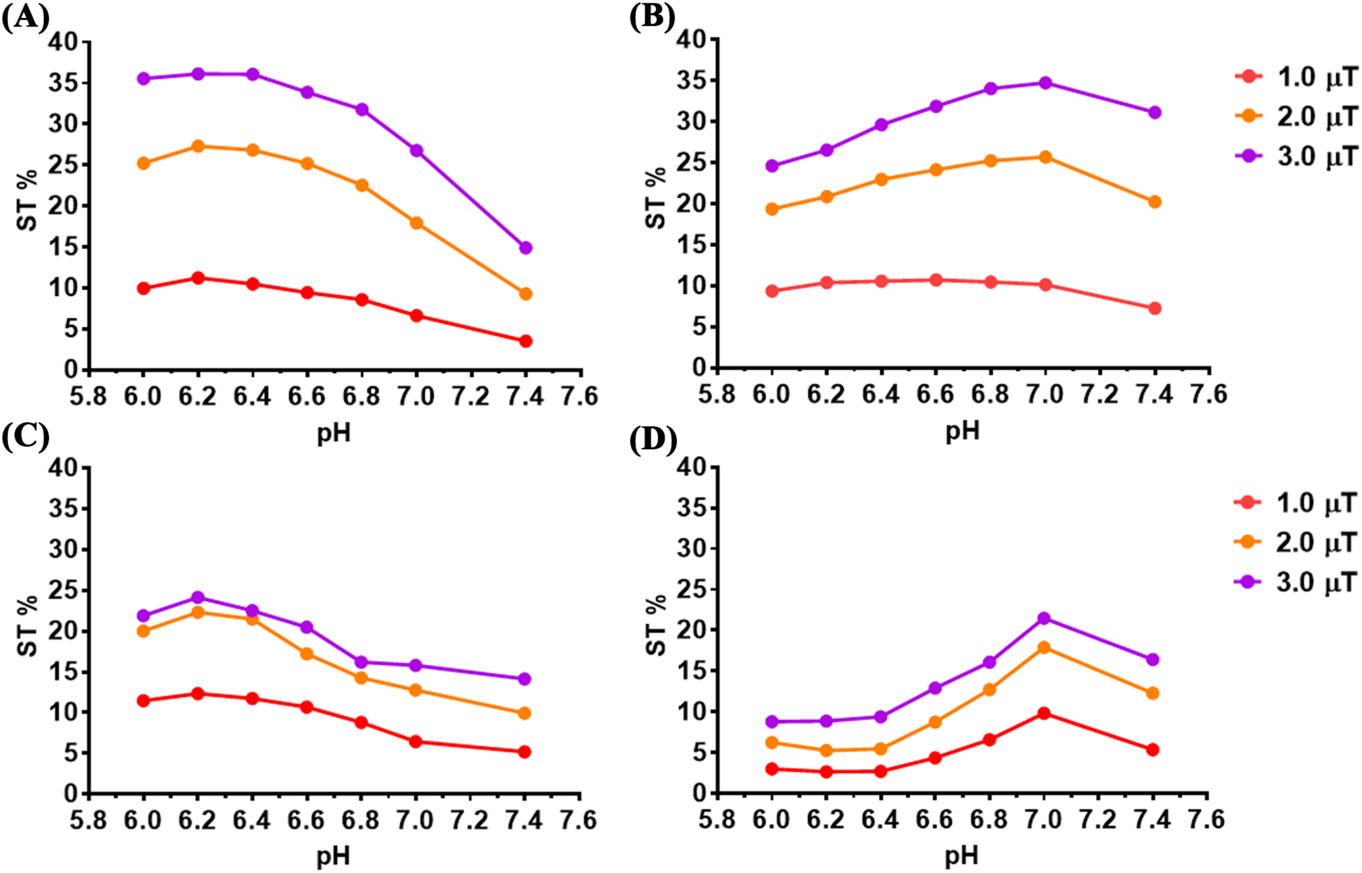
pH and B1 power dependence for the two chemical exchange saturation transfer (CEST) agents. Percentage change in saturation transfer (ST%) effect plot of 20 mM (A and C) D‐Glucose (calculated at 0.8 ppm) and of (B and D) 3‐O‐Methyl‐D‐glucose (3OMG) (calculated at 1.2 ppm) solutions containing 10 mM of phosphate buffer as a function of the RF saturation field (B_1_ = 1.0, 2.0 and 3.0 μT) acquired at (A and B) 7 T and (C and D) 3 T and 37°C

Figure 3 reports the CEST contrast measured at 37°C and 7 or 3 T, at a frequency offset of 0.8 ppm for D‐Glucose and 1.2 ppm for 3OMG. From these data, there are useful insights concerning the pH dependence for the two CEST agents at different saturation power values (1.0, 2.0 and 3.0 μT). For D‐Glucose, an increase in the CEST effect at pH 6.0–6.2 was observed for all the selected saturation power values (Figure 3A). The effect was more pronounced using a 3.0‐μT power pulse and decreased proportionally with increasing pH, reaching a minimum at pH 7.4. In the case of 3OMG, compared with D‐Glucose an opposite trend was observed (Figure 3B): the CEST response increased slightly, starting from an acidic pH value up to pH 7.0, followed by a slight decrease at pH 7.4. At a lower B_0_ field, the irradiation RF field of 3.0 μT resulted in the highest CEST contrast response for both D‐Glucose and 3OMG. D‐Glucose still displayed a higher CEST effect at acidic pH values, but the CEST contrast at 2.0 and at 3.0 μT were almost comparable (Figure 3C). For 3OMG the detected CEST effect showed a sharp increase from pH 6.4 that peaked at pH 7.0 then decreased (Figure 3D).

### 3.2 In vivo CEST MRI studies

**FIGURE 4:**
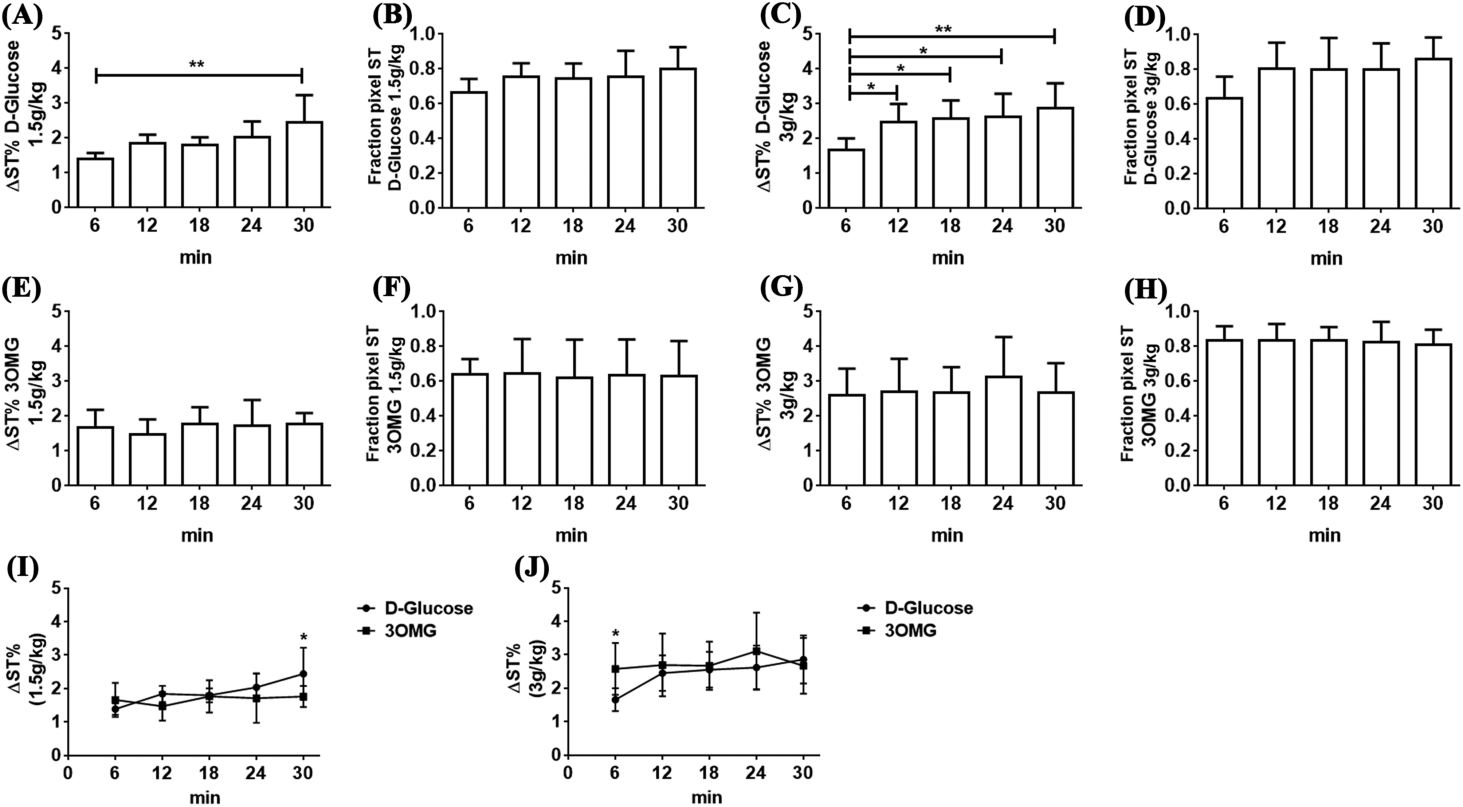
A marked increase in chemical exchange saturation transfer (CEST) contrast in the tumor region was observed at 7 T following intravenous injection of D‐Glucose or 3‐O‐Methyl‐D‐glucose (3OMG). GlucoCEST contrast was obtained with D‐Glucose at doses of (A) 1.5 or (C) 3 g/kg and with 3OMG at doses of (E) 1.5 or (G) 3 g/kg, as well as the fractions of enhanced pixels for (B and D) D‐Glucose and (F and H) 3OMG at the corresponding injected doses. Data are reported as the percentage change in saturation transfer effects (ΔST%) at 1.2 ppm before and after the injection. Also shown are CEST contrast (ΔST%) against time curves for D‐Glucose and 3OMG administered at doses of (I) 1.5 and (J) 3 g/kg

To determine the minimum dose needed to obtain sufficient glucoCEST contrast, mice inoculated with B16‐F10 melanoma cells on both flanks underwent i.v. administration at doses of 1.5 or 3 g/kg of D‐Glucose or 3OMG. Precontrast and postcontrast images were acquired with 7‐ and 3‐T preclinical scanners by applying a 2.0‐μT RF pulse for 5 s and CEST contrast was calculated at 1.2 ppm for both molecules. At 7 T, as shown in Figure 4A, the CEST contrast reached a value of 1.4% ± 0.2% 6 min after a single bolus glucose i.v. injection of 1.5 g/kg. The CEST contrast slightly increased to reach a value of 2.4% ± 0.7% 30 min after the injection. The calculated fraction of enhanced pixels (i.e. pixels showing a positive ΔST increase; Figure 4B) indicates good coverage of the tumor region (from 66% right after the injection to 78% after 30 min). A higher single D‐Glucose dose of 3 g/kg improved the CEST signal over 30 min of observation, starting from a CEST contrast of 1.7% ± 0.3% that increased to 2.9% ± 0.7% (Figure 4C). Almost 80%–85% of the tumor area showed a marked glucoCEST enhancement 12 min after the D‐Glucose injection (Figure 4D). ANOVA analysis indicated a significant difference in CEST contrast from 6 min after the injection to 12, 18, 24 or 30 min later (*p* = 0.046, 0.022. 0.013 and 0.0016, respectively) at the highest dose and from 6 to 30 min for the lower dose (*p* = 0.0011).

**FIGURE 5:**
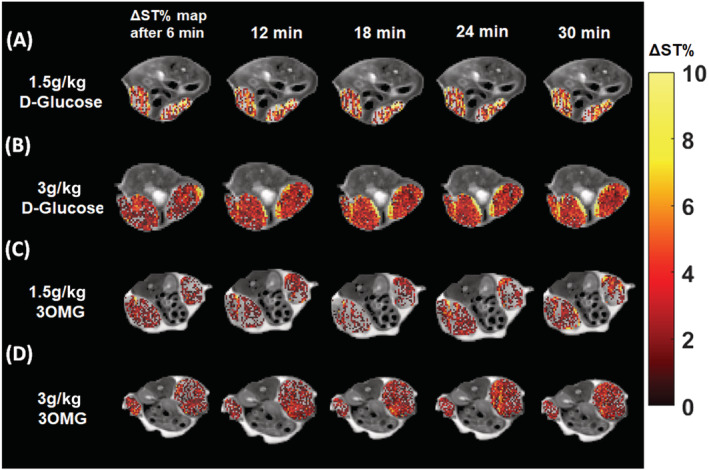
D‐Glucose chemical exchange saturation transfer (GlucoCEST) percentage change in saturation transfer (ΔST%) map obtained at 7 T upon intravenous injection of D‐Glucose at doses of (A) 1.5 and (B) 3 g/kg or 3‐O‐Methyl‐D‐glucose (3OMG) at (C) 1.5 and (D) 3 g/kg. Data are reported as the ΔST% at 1.2 ppm before and every 6 min after the injection. Parametric maps are overimposed to T_2w_ anatomical images and glucoCEST contrast is shown only in the tumor regions

Conversely, the 3OMG CEST contrast remained stable over the 30‐min observation time, but was comparable in magnitude with that raised by D‐Glucose (Figure 4E,G). 3OMG at the 1.5 g/kg dose showed a ΔST% of 1.7% ± 0.5% and a fraction of enhanced pixels of ~ 62%–64% (Figure 4E,F). The 3 g/kg dose injection showed a marked increase in the CEST response (ΔST% 2.7% ± 0.9%), with a fraction of enhanced pixels of 80%–83% (Figure 4G,H). Comparison of the CEST contrast against time curves showed similar increases in ΔST% for glucose and 3OMG at both doses (Figure 4I,J). Representative parametric images for both D‐Glucose and 3OMG at the two doses and at several time points (6–30 min after i.v. administration) are shown in Figure 5.

**FIGURE 6:**
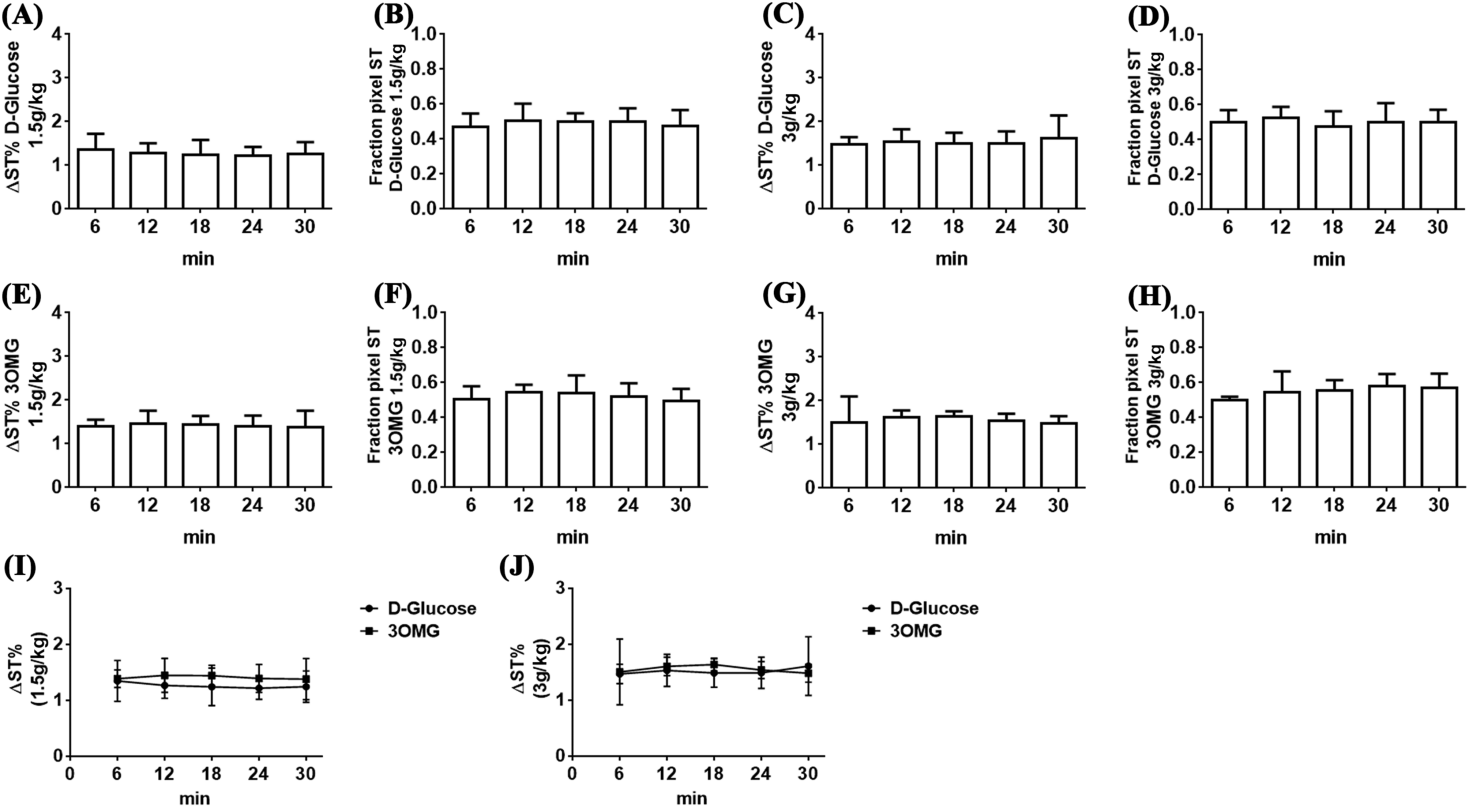
A modest increase in chemical exchange saturation transfer (CEST) contrast in the tumor region was observed at 3 T following intravenous injection of D‐Glucose or 3‐O‐Methyl‐D‐glucose (3OMG). GlucoCEST contrast was obtained with D‐Glucose at doses of (A) 1.5 or (C) 3 g/kg and with 3OMG at doses of (E) 1.5 or (G) 3 g/kg, as well as the fractions of enhanced pixels for (B and D) D‐Glucose and (F and H) 3OMG at the corresponding injected doses. Data are reported as the percentage change in saturation transfer effects (ΔST%) at 1.2 ppm before and after the injection. Also shown are CEST contrast (ΔST%) against time curves for D‐Glucose and 3OMG administered at doses of (I) 1.5 and (J) 3 g/kg doses

At 3 T, both D‐Glucose and 3OMG displayed a smaller CEST contrast. The ΔST% of D‐Glucose was 1.3% ± 0.3% from a bolus injection of 1.5 g/kg (Figure 6A) and 1.5% ± 0.3% from a bolus injection of 3 g/kg (Figure 6C), and it remained substantially stable over time. Moreover, the fraction of enhanced pixels displayed a similar percentage of 47%–50% for the 1.5 g/kg dose and 50%–52% for the 3 g/kg dose (Figure 6B,D).

**FIGURE 7:**
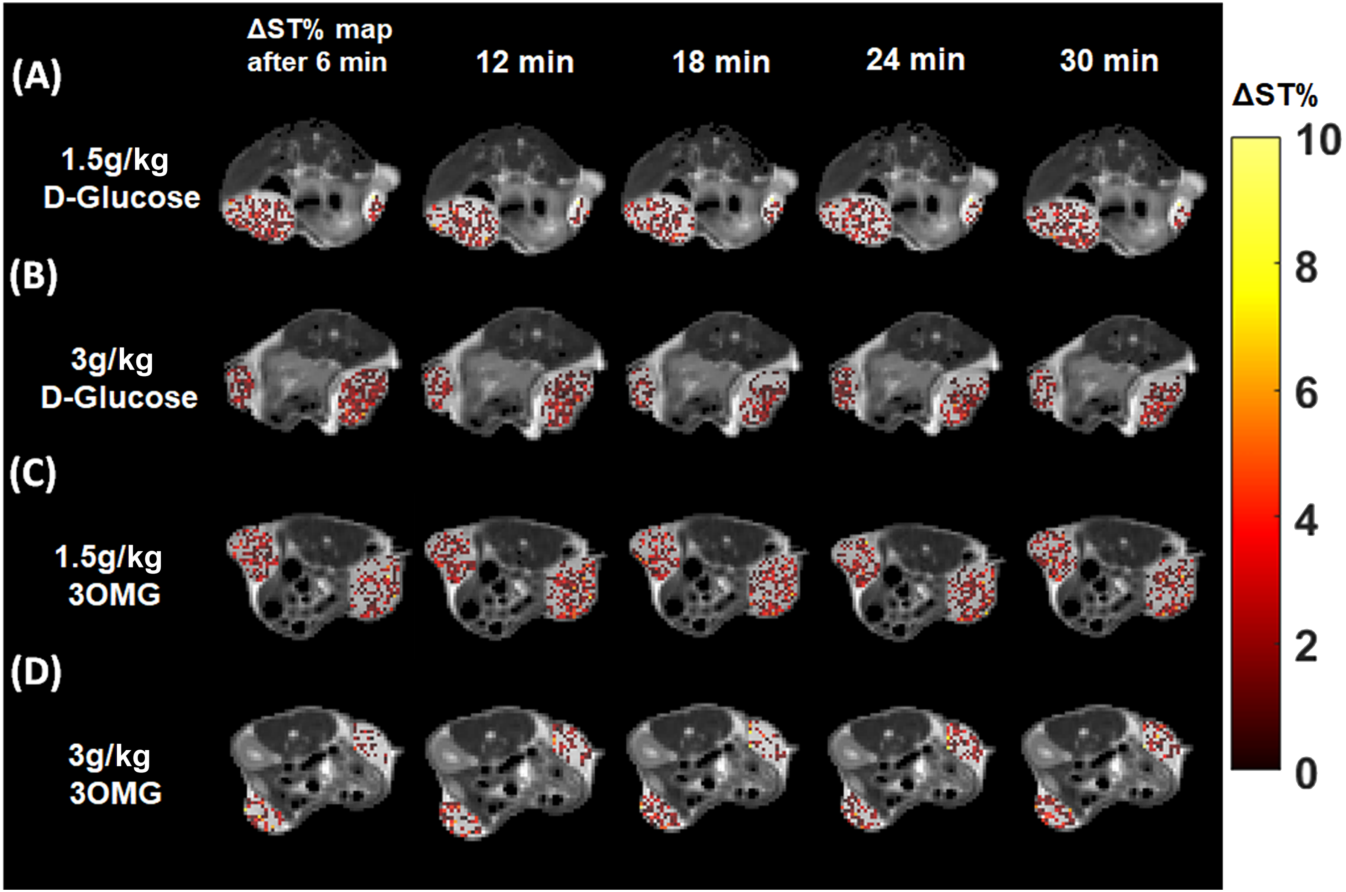
D‐Glucose chemical exchange saturation transfer (GlucoCEST) percentage change in saturation transfer (ΔST%) map obtained at 3 T after intravenously injecting D‐Glucose at doses of (A) 1.5 and (B) 3 g/kg or 3‐O‐Methyl‐D‐glucose (3OMG) at doses of (C) 1.5 and (D) 3 g/kg. Data are reported as ΔST% at 1.2 ppm before and every 6 min after the injection. Parametric maps are overimposed to T_2w_ anatomical images and glucoCEST contrast is shown only in the tumor regions

Comparable CEST contrast enhancements and fractions of enhanced pixels were observed for 3OMG at both doses. At the dose of 1.5 g/kg, ΔST% of 1.4% ± 0.2% was observed, whereas at the dose of 3 g/kg, ΔST% was 1.6% ± 0.2%, and remained stable for 30 min (Figure 6E,G). Almost similar fractions of enhanced pixels were observed for both doses: 49%–53% for 1.5 g/kg and 50%–56% for 3 g/kg (Figure 6F,H). At lower field, when comparing the two molecules at the same dose, any difference in glucoCEST contrast was detectable (Figure 6I,J). Representative parametric images for both D‐Glucose and 3OMG at the two doses and several time points (6–30 min after i.v. administration) are shown in Figure 7, depicting a clear reduction in the CEST contrast and in the coverage of the enhanced pixels inside the tumor, for both the molecules at 3 T, and in comparison with 7 T.

## 4 DISCUSSION AND CONCLUSIONS

Overall, the results reported herein highlight the role of D‐Glucose and 3OMG as CEST agents for tumor detection. Both in vitro and in vivo results outline the better performance of these agents at higher magnetic field strength, due to the larger chemical shift separation between hydroxyl and water resonances. At the lower magnetic field examined (3 T, a field strength available on clinical scanners), the separation in Hz between the hydroxyl and the bulk water protons is reduced, thus increasing the direct saturation of the bulk water pool that may decrease the labeling efficiency of the hydroxyl protons and consequently reduce the contrast performance. The CEST effect in both molecules was smaller at lower B_0_ as expected.

The main difference between the CEST properties of D‐Glucose and 3OMG that emerged from the in vitro investigations concerns the pH dependence. In the pH range of 6–7.4, the extent of glucoCEST contrast for D‐Glucose was higher moving towards the acidic side, whereas 3OMG displayed higher ST values when pH was close to neutrality, as already noted in vitro by examining the single compounds.^15^, ^29^

Although surprising, this different pH dependence can be explained by considering the slower exchange rate values for the hydroxylic protons of 3OMG in comparison with glucose.^21^, ^38^ Additionally, because of the mutarotation process in which both the molecules are involved, the ratio of α and β anomers at equilibrium is different between 3OMG (β:α = 58:42) and glucose (β:α = 64:36).^39^, ^40^ This different ratio might affect the exchange rate, because the β anomer shows faster exchange rates.^41^

In an in vivo setting, single i.v. bolus injections of either D‐Glucose or 3OMG at 7 T yielded well‐detectable CEST effects in the tumor region (dose 3 g/kg, max. CEST values of ~ 2%–3% for D‐Glucose and 3OMG). Conversely, at the magnetic field of 3 T, the average CEST effect for both contrast agents was slightly smaller but still above 1%. Consequently, at the higher magnetic field strength of 7 T, a detectable difference between the two doses was observed, whereas at the lower magnetic field strength (3 T), a clear difference between the two doses was not detected. A similar decrease in the theoretically achievable glucoCEST contrast between 7 and 3 T was recently reported from simulated data in a tumor‐like tissue,^21^ and a small if not elusive CEST contrast was detected in cancer patients following glucose administration at 3 T.^42^, ^43^ Our results are in line with those reported in the literature, despite the different experimental conditions (including tumor models and perfusion that affect exogenous contrast agent accumulation, B_0_ field, B_1_ saturation level and duration, administration route and doses, as well as image analysis) that hamper a robust comparison among studies and between molecules. For example, Chan et al. measured a glucoCEST contrast of 3%–4% after i.v. infusion of D‐Glucose (dose of 1 g/kg, 11.7 T) in tumor‐bearing mice,^15^ and at the same B_0_ field using a dynamic glucose enhanced MRI (DGE‐MRI) approach, a 1% enhancement in the tumor region was observed 5 min after injection in a brain tumor model with a dose of 3 g/kg.^44^ For 3OMG, Rivlin and Navon reported a glucoCEST contrast of ~ 4% 30 min after the i.v. injection of 3OMG (dose of 0.7 g/kg) on implanted orthotopic mammary tumors at 7 T,^32^ whereas, in a brain tumor model, Sehgal et al. reported a contrast of around 3% (with a dose of 3 g/kg at 11.7 T) during DGE acquisition.^45^ For the first time, the current study provides a strict comparison in terms of the glucoCEST contrast achievable by the two investigated molecules at different magnetic fields.

Detection of CEST agents requires the accumulation in the ROI of an enough concentration (of the order of mM) of exchangeable protons. Both dose regimens used in this work allowed the detection of the associated CEST effect in a large portion of the tumor region, with higher fractions of enhanced pixels when increasing the dose or the main magnetic field. Concerning the administration of the molecules, we only investigated the i.v. injection route because it provides faster penetration of the agents into the tumor region and is the most common administration route for MRI contrast agents at clinical level.

Upon i.v. injection, D‐Glucose and 3OMG distribute in the systemic circulation and maintain high concentration levels in the tumor region. The main difference between the two agents is related to their metabolic fate: both are taken up by the tumor cells but only D‐Glucose is metabolized, whereas 3OMG is not. On this basis, it was surmised that 3OMG, through its accumulation in the tumor cell, may act as a reporter of the glucose transporters, similar to what occurs with [^18^F]‐FDG in PET examinations. Consequently, 3OMG could be exploited not only for detecting tumors, but also for monitoring tumor progression and response to therapy.

The behavior of the CEST effects detected over the 0–30 min observation time could reflect, as well as variations in concentrations associated with tumor‐perfusion properties,^46^ changes in the pH of the microenvironments in which the agents are distributed. Considering the CEST effect shown by 3OMG (Figure 4G), the observed response may arise from the extracellular contribution (that decreases upon time, characterized by an acidic pH, thus yielding a smaller ST%, as shown by the in vitro experiments) and the intracellular contribution (that increases upon time, characterized by a higher ST%, as shown by the in vitro experiments). Thus, the constancy shown over almost all of the 30‐min observation time could be the balanced result of the two contributions. Vice versa, in the case of D‐Glucose, the marked increase in the ST% (Figure 4C) strongly suggests a pH decrease that occurs in the extracellular matrix of the tumor cells, despite some of the molecule being metabolized inside the cancer cells. In fact, several previous studies demonstrated that higher glucose dose administrations are exploited by cancer cells in increased aerobic glycolysis that results in lactic acid production. Consequently, tumor cells exploit several proton transporters to remove lactate and acid equivalents from cytosol to the extracellular space to maintain a neutral pH. This will induce an extracellular acidification that leads to a decrease in the extracellular pH.^47^, ^48^, ^49^ Therefore, the decrease in the extracellular pH could be responsible for the observed enhanced ST%, as is clearly shown in the in vitro results, where D‐Glucose yielded an improved CEST performance at more acidic pH values.

Further improvements of the glucoCEST approach are ongoing. For instance, aiming at improving glucose irradiation specificity and reducing direct water saturation effects for an enhanced glucoCEST detection, application of the CESL technique was proposed. Jin et al.^50^ reported robust glucoCESL signal at 9.4 T using 0.25 g/kg of glucose. Zu et al.,^51^ at the same field strength, for the 3OMG i.v. injection (dose 1.5 g/kg), reported increased signal in a rat brain tumor model compared with healthy brain by appropriate analyses of MRI signals. Development of adiabatically prepared spin‐lock sequences allowed the implementation of the glucoCESL approach in clinical scanners with better visualization of glucose uptake.^22^, ^23^, ^35^ Recently, Zaiss et al. provided an accurate procedure for the optimization of the acquisition parameters in vivo based on a detailed study of the glucose‐OH exchange rate under physiological conditions.^21^ An ultrahigh‐field scanner at 17.2 T can be used to investigate the metabolic changes induced by neuronal stimulation in rat brain with the GlucoCEST technique.^52^

To the best of our knowledge, this is the first study in which D‐Glucose and 3OMG have been systematically compared in terms of their CEST contrast efficiency at different magnetic fields and administered doses in the same murine tumor model. Further investigations are needed to compare the glucose and 3OMG CEST response with the [^18^F]‐FDG PET technique to assess its potential as a valid alternative for tumor diagnosis and treatment monitoring.

## FUNDING INFORMATION

We acknowledge the support received from the European Union's Horizon 2020 research and innovation programme (grant agreement no. 667510), from the Associazione Italiana Ricerca Cancro (AIRC MFAG 2017; ID: 20153 project ‐ P.I. Dario Livio Longo) and from the Compagnia San Paolo project (Regione Piemonte, grant #CSTO165925). MC was supported by a fellowship from the Associazione Italiana Ricerca Cancro (AIRC ID 24104). The Italian Ministry for Education and Research (MIUR) is gratefully acknowledged for yearly FOE funding to the Euro‐BioImaging Multi‐Modal Molecular Imaging Italian Node (MMMI).
